# Recent advances in the epidemiology, diagnosis, and management of *Trichomonas vaginalis* infection

**DOI:** 10.12688/f1000research.19972.1

**Published:** 2019-09-20

**Authors:** Olivia T. Van Gerwen, Christina A. Muzny

**Affiliations:** 1 Division of Infectious Diseases, University of Alabama at Birmingham, Birmingham, AL, 35294, USA

**Keywords:** Trichomonas vaginalis, vaginal infections, STI

## Abstract

*Trichomonas vaginalis *is the most common, curable non-viral sexually transmitted infection (STI) worldwide. Despite this burden of disease, it is not currently a reportable disease in the United States. Recent advances in the epidemiology, diagnosis, and management of *T. vaginalis* infection are described in this article. This includes updated global and U.S. prevalence data in women and men as well as recent epidemiological data in HIV-infected individuals and pregnant women. Advances in molecular diagnostics are also reviewed, as are data from recent clinical trials regarding the treatment of trichomonas in women.

## Introduction

*Trichomonas vaginalis* is the most common, non-viral sexually transmitted infection (STI) worldwide ^1^. Patients with symptomatic *T. vaginalis* report various symptoms, including vaginal discharge and dysuria in women and urethral discharge and dysuria in men ^2– 4^. Many infected patients, however, never experience symptoms ^3^. Untreated or persistent *T. vaginalis* in women has been associated with infertility and adverse birth outcomes ^5– 7^. While less is known about *T. vaginalis* in men, it has been described as a cause of nongonococcal urethritis (NGU), prostatitis, and epididymitis ^4^. *T. vaginalis* has also been linked to an increased risk of HIV, posing a major public health threat ^8^. Nevertheless, *T. vaginalis* remains understudied given the lack of public health attention it has received ^9^. It is not currently a reportable disease in the U.S., as it has previously been found to meet only three out of seven criteria ^10^. This review aims to provide an update on recent advances in the epidemiology, diagnosis, and treatment of *T. vaginalis*.

## Epidemiology

The World Health Organization (WHO) estimated 156 million cases of *T. vaginalis* worldwide in 2016, accounting for almost half of the global STI incidence that year ^1^. Updated epidemiological data on the national prevalence of trichomonas among women and men in the U.S. was published in 2018 ^11^. These data were collected during 2013–2014 in the National Health and Nutrition Examination Survey (NHANES) using the Hologic Gen-Probe Aptima® *T. vaginalis* assay on urine specimens. *T. vaginalis* prevalence was 1.8% in women and 0.5% in men aged 18–59 years. Prior to this study, the national prevalence of *T. vaginalis* had been poorly characterized among U.S. men as a result of diagnostic challenges; NHANES did not test men for *T. vaginalis* until 2013–2014. Spontaneous resolution of *T. vaginalis* is known to occur at relatively high rates (36–69%) in men ^12, 13^, which may explain the lower prevalence compared to women in the recent NHANES study. While *T. vaginalis* is less common in men ^11^, it is readily passed between sexual partners during penile–vaginal sex, even when the infected partner is asymptomatic. Thus, treatment of infected men is an important public health concern ^14^. In men who have sex with men (MSM), *T. vaginalis* rarely causes urethral or rectal infection, and screening in asymptomatic individuals is of low utility ^15^.

A marked racial disparity regarding *T. vaginalis* was noted among African American women and men in the recent NHANES study, with an estimated prevalence of 6.8% among the black population compared to 0.4% among other groups ^11^. This is consistent with 2001–2004 NHANES data which found higher rates of *T. vaginalis* in African American women compared to women of other races/ethnicities ^16^. This pronounced racial disparity is likely multifactorial, involving differences in sexual networks, individual-level sexual risk behaviors such as larger numbers of sexual partners, and structural disparities (i.e. inadequate access to healthcare resources) ^17– 19^.

In the recent NHANES study, *T. vaginalis* was found to be significantly associated with older age, lower educational level, lower socioeconomic status, and having two or more sexual partners in the past year ^11^. Compared to other high-income countries such as the United Kingdom, *T. vaginalis* prevalence in the U.S. is higher, possibly a function of the lack of public health consideration it receives ^20^.

The only population for which routine *T. vaginalis* screening is currently recommended in the U.S. is HIV-infected women ^21^. Even in the absence of symptoms, *T. vaginalis* in this population has been associated with high rates of adverse events such as pelvic inflammatory disease (PID) and poor birth outcomes ^5, 22^. Several recent studies have found high *T. vaginalis* prevalence (17.4–20%) ^23– 26^ and repeat infection rates (up to 22.7% over a median of 16 months) among HIV-infected women ^23^. Similar to HIV-uninfected men, *T. vaginalis* is less common in HIV-infected men and infrequently seen in HIV-infected MSM ^24^.

Since *T. vaginalis* has been associated with adverse birth outcomes, its effect on pregnant women is important to consider ^6^. Currently, there are no recommendations for screening of asymptomatic pregnant women for *T. vaginalis*. This is largely owing to results from a prior randomized controlled trial (RCT) of asymptomatic pregnant women with *T. vaginalis* ^27^. This study found a higher risk of preterm delivery among women treated with two doses (2 grams each) of metronidazole (MTZ) 48 hours apart between 16 and 23 and between 24 and 29 weeks of gestation, respectively, versus placebo. However, this trial had several limitations including atypical MTZ dosing. In addition, the second round of MTZ was given between 24 and 29 weeks’ gestation, whereas the greatest increase of pre-term delivery was at 35–36 weeks in this study. Because of this, definitive conclusions regarding an association between MTZ treatment of asymptomatic *T. vaginalis* during pregnancy and pre-term birth cannot be made and further studies are needed ^27, 28^.

The global prevalence of *T. vaginalis* in pregnant women varies geographically. A 2016 systematic review of 75 studies of STI prevalence among pregnant women found that *T. vaginalis* prevalence ranged from 3.9–24.6% in low- to middle-income countries (i.e. Latin America and Southern Africa) ^29^. Recent studies found a *T. vaginalis* prevalence of 20% among HIV-infected pregnant women in South Africa ^26^ as well as high rates of incident infection in pregnant women (9.2/100 person-years) in South Africa and Zimbabwe ^30^. Unexpectedly high rates of persistent *T. vaginalis* (44% at 21 days or more following treatment) by nucleic acid amplification test (NAAT) in pregnant women in the Southern U.S. have been noted by Lazenby *et al*. ^31^. This is higher than the rate of 7% in pregnant women noted in a previous U.S. study ^27^. Based on their data, Lazenby *et al*. suggested that all pregnant women with *T. vaginalis* should be re-tested with NAATs approximately 3 weeks post-treatment ^31^.

## Diagnosis

The primary diagnostic modality for *T. vaginalis* has traditionally been microscopic examination of a wet mount of vaginal fluid, looking for motile trichomonads ^32^. While wet mount is inexpensive and rapid, its use is limited by low sensitivity, which ranges from 44–68% compared to culture ^33^. Prior to the advent of NAATs, culture was the gold standard for diagnosis of *T. vaginalis*, with a sensitivity of 81–94% ^34– 36^. Diamond’s medium is the traditional culture method used for the isolation of *T. vaginalis* ^37^. However, contamination with vaginal bacteria is common, making this technique difficult ^34^. Culture systems, such as the InPouch® system (BioMed Diagnostics, White City, OR), have been developed to avoid contamination by placing the specimen in a two-chambered bag, allowing for simultaneous sampling for wet mount and culture while maintaining similar efficacy to Diamond’s medium ^38^.

Despite relatively high sensitivity and specificity, *T. vaginalis* culture remains time consuming, as it requires incubation and reading of the InPouch® over several days ^39^. It is categorized by the Clinical Laboratory Improvement Amendments (CLIA) as moderately complex ^40^. Over the past several years, molecular testing for *T. vaginalis* has become the preferred diagnostic modality ^21^. There are three US Food and Drug Administration (FDA)-approved *T. vaginalis* NAATs currently available in the U.S., all of which are able to detect co-infection with gonorrhea and chlamydia from the same specimen ^33, 41– 43^. The Aptima® *T. vaginalis* assay (Hologic, Bedford, MA) was the first NAAT to be approved by the FDA for *T. vaginalis* detection in asymptomatic and symptomatic women ^41^. This assay detects an rRNA target via transcription-mediated amplification (TMA), with sensitivity ranging from 88–100% and specificity from 98–100% ^44^. It can be performed on clinician-obtained vaginal and endocervical swab specimens, urine specimens, and ThinPrep PreservCyt specimens with results in less than 8 hours ^41^. Importantly, it has not been FDA approved for use in men and must be internally validated before use ^33^. The BD ProbTec *T. vaginalis* Q ^x^ (TVQ) amplified DNA assay (BD Diagnostics, Baltimore, MD) was the second *T. vaginalis* NAAT approved by the FDA for use in female urine, endocervical swab specimens, and patient- or clinician-obtained vaginal specimens ^42^. Similar to the Aptima® TV assay, this test is only FDA approved in women and must be internally validated prior to use in men. The TVQ assay is able to yield results in less than 8 hours ^42^.

The Xpert® TV assay (Cepheid, Sunnyvale, CA) was the first *T. vaginalis* NAAT FDA approved for use in female urine, endocervical swab, and patient- and clinician-collected vaginal specimens as well as male urine ^43^. Diagnostic sensitivity and specificity for the Xpert® TV assay range from 99.5–100% and 99.4–99.9% for female genital specimens and 97.2–99.9% for male urine specimens ^43^. Once collected and placed in the testing platform, the Xpert® TV assay yields results in 60–90 minutes, allowing for point-of-care (POC) diagnosis and management ^45^.

POC STI diagnostics are powerful tools, allowing providers to accurately diagnose and provide appropriate treatment for patients in the same visit. Given the communicability and frequency of *T. vaginalis*, rapid diagnosis and treatment could have a substantial impact on its public health burden ^46^. The Solana® Trichomonas assay (Quidel, San Diego, CA) is a new rapid test for the qualitative detection of *T. vaginalis* DNA and can yield results within 40 minutes of specimen collection ^47^. This assay was FDA approved in 2017 for the diagnosis of *T. vaginalis* from female vaginal and urine specimens from asymptomatic and symptomatic women, with sensitivity compared to NAAT of over 98% for vaginal specimens and over 92% for urine ^47^. Solana® requires a specific testing instrument to process samples; thus, similar to NAATs, there is upfront cost associated with its use. After a specimen is collected, it is lysed by heat, diluted, and added to a reaction tube containing helicase-dependent amplification (HDA) reagents including primers specific for the amplification of a *T. vaginalis*-specific target sequence ( https://www.quidel.com/molecular-diagnostics/solana-trichomonas-assay). The AmpliVue® Trichomonas Assay (Quidel, San Diego, CA) is another POC test providing qualitative detection of *T. vaginalis* which is FDA approved for vaginal specimens from symptomatic and asymptomatic women ^48^. Similar to Solana®, AmpliVue® also uses HDA technology but testing can be performed in a small handheld cartridge that requires no additional equipment ( http://www.quidel.com/molecular-diagnostics/amplivue-products). AmpliVue® has demonstrated comparable sensitivity and specificity to the Aptima® TV assay, at 90.7% and 98.9%, respectively ^48^.

The OSOM® Trichomonas Rapid Test (Sekisui, Framingham, MA) is a qualitative antigen-detection immunochromatographic assay with a processing time of 10–15 minutes ^49^. It is validated for the diagnosis of *T. vaginalis* in women from clinician-obtained vaginal specimens with a sensitivity of 83–92% and specificity of 99% ^50, 51^. This test has not performed well in identifying infection in male urine when compared to the Aptima® TV assay ^51^; thus, it is currently recommended in women only ^21^. Because it requires no special equipment and is low cost, the OSOM® test is appealing in the setting of STI testing campaigns in low-resource settings ^52^.

With the advent of these numerous high-quality *T. vaginalis* diagnostics, clinicians should be aware of which of the above tests are currently available in their laboratories beyond wet mount. In choosing which of the above diagnostic tests to use, the need for a rapid diagnosis should be balanced with the need for a highly sensitive test. Furthermore, there are several additional trichomoniasis diagnostic tests in the pipeline whose performance characteristics have not yet been published.

## Management

The management of trichomoniasis continues to evolve, particularly in women. Per the 2015 Centers for Disease Control and Prevention (CDC) sexually transmitted disease treatment guidelines ^21^, first-line treatment for trichomoniasis in HIV-negative women and men includes a single 2 gram dose of oral MTZ or tinidazole (TIN). Oral MTZ 500 mg twice daily for 7 days is an alternative therapy. These recommendations were based on several small trials conducted over 30 years ago ^53– 56^, several of which had a high potential for bias, as well as a desire to reduce adherence issues with multi-dose therapy. A meta-analysis of prior trichomonas treatment trials recently showed that women receiving the 7-day regimen of oral MTZ were 50% less likely to be positive at test of cure (TOC) compared to those receiving single-dose therapy ^57^. In addition, RCTs of HIV-infected women with *T. vaginalis* ^58^ and HIV-negative women with *T. vaginalis* ^59^ found that the 7-day MTZ dose was more effective than single-dose therapy at TOC. While the CDC recommends only 7-day MTZ for HIV-infected women, it is highly likely that this regimen will be recommended for all women moving forward ^60^. Of note, a recent literature review found no increased risk of teratogenicity with the use of MTZ (a class B drug) during pregnancy ^61^. TIN has not been evaluated in pregnancy and remains a class C drug.

There have been no head-to-head comparisons of the single 2 gram dose of oral MTZ and the 7-day regimen in men. One observational study of 325 men with trichomoniasis found that treatment with the 2 gram dose of oral MTZ was unsuccessful in 42.9% of cases ^62^. A second study found that the microbiological efficacy of treatment with the 2 gram dose of oral MTZ in men was 77.1% ^63^. While these cure rates seem to be suboptimal, neither of these studies had a comparison group with the 7-day MTZ regimen; thus, no conclusions can be made.

In addition to MTZ, TIN is another 5-nitroimidazole medication that is FDA approved and recommended by the CDC for the treatment of trichomoniasis. TIN has better absorption and fewer gastrointestinal side effects than MTZ ^64^ but is ten times more expensive (with an approximate retail price of $44.66 per 2 gram dose, compared with $3.47 per 2 gram dose of MTZ at the time of writing) ^3^ and less likely to be adopted by clinicians. Other nitroimidazoles, such as secnidazole ^65^ and ornidazole ^63, 66^, have been used in other countries but are not currently FDA approved for the treatment of trichomoniasis in the U.S.

Persistent or recurrent infection due to antimicrobial-resistant *T. vaginalis* or other causes should be differentiated from reinfection from an untreated or insufficiently treated sexual partner. A detailed patient history should be taken to assess the likelihood of reinfection. Following treatment failure, and if reinfection has been excluded, persistent or recurrent trichomoniasis ^3^ has been treated successfully with longer courses or additional doses of the same medications used in standard therapy (i.e. high-dose oral MTZ or TIN 2 grams orally daily for 7 days) ^21^. Single-dose MTZ or TIN therapy should be avoided ^21^. If drug resistance is suspected, the isolate can also be sent to the CDC for drug resistance testing ( https://www.cdc.gov/laboratory/specimen-submission/detail.html?CDCTestCode=CDC-10239). If the patient fails the 7-day regimen of high-dose oral MTZ or TIN, two additional treatment options are available which have had successful results. The first is high-dose oral TIN 2–3 grams daily (in divided doses) plus intravaginal TIN 500 mg twice daily for 14 days ^67^. The second is high-dose oral TIN (1 gram three times daily) plus intravaginal paromomycin (4 g of 6.25% intravaginal paromomycin cream nightly) for 14 days ^68, 69^. Paromomycin is an aminoglycoside with a different mechanism of action (destruction of ribosomal RNA) than MTZ (inhibition of nucleic acid synthesis by DNA disruption) ^70^; successful treatment of MTZ-resistant trichomoniasis with both high-dose oral TIN and intravaginal paromomycin may suggest a synergistic effect. It should be noted, however, that intravaginal paromomycin cream may cause vaginal ulceration(s) ^71^. These ulcerations may spontaneously regress when therapy is stopped. Expert consultation should be sought for additional treatment options for patients who fail the above treatment options. There are currently no data regarding optimal treatment of male sexual partners of women with MTZ-resistant trichomoniasis.

Another complicated treatment scenario for women with trichomoniasis is the setting of an IgE-mediated-type hypersensitivity reaction to 5-nitroimidazoles. These patients should be managed by MTZ desensitization according to a published regimen ^72, 73^ and in consultation with an allergy specialist ^21^. Treatment of patients with *T. vaginalis* who are unable to be desensitized is difficult and mainly based on anecdotal data ^21, 74^. One option for which we have had success is intravaginal boric acid 600 mg twice daily for 60 days ^75, 76^.

## Partner treatment

Per current CDC sexually transmitted disease treatment guidelines, concurrent treatment of all sexual partners of patients with trichomoniasis is critical for symptomatic relief, microbiologic cure, and prevention of transmission and reinfection ^21^. In the last several years, the evidence for expedited partner therapy (EPT) as a mechanism of prevention of *T. vaginalis* infection has grown ^77^. EPT, or the treatment of sexual partners of a patient diagnosed with an STI by providing treatment prescriptions to the patient without clinical assessment of the partners ^78^, has been recognized as an effective option for partner treatment of chlamydia and gonorrhea ^79^. The CDC currently recommends EPT as an option for partner therapy for these STIs in women and heterosexual men ^21, 79^. Interestingly, two prior RCTs conducted on the use of EPT in partners of women with trichomoniasis had mixed results ^80, 81^. Schwebke *et al*. found EPT to be well accepted and safe with rates of repeat infection in the EPT arm lower than those of the public health disease intervention and partner-referral arms ^81^. In an RCT by Kissinger *et al*., however, randomization to *T. vaginalis* EPT did not lead to increased partner treatment uptake or lower follow-up rates compared to standard partner referral ^80^. Nevertheless, given high reinfection rates and frequent asymptomatic infection in men, EPT is still recommended as a valid means of partner therapy of *T. vaginalis*-infected patients ^21^. EPT has also recently been shown to be effective in decreasing repeat *T. vaginalis* infection rates when used in conjunction with POC testing strategies in South African women ^52^. Despite some data supporting EPT for patients with *T. vaginalis*, implementation has been limited because of legal restrictions in many states ( https://www.cdc.gov/std/ept/legal/default.htm). Additionally, EPT acceptance has been found to vary with patient and provider characteristics ^82^. It is currently unknown how often providers prescribe EPT for trichomoniasis in the U.S.

## Conclusion

In recent years, many advances have been made in the epidemiology, diagnosis, and treatment of *T. vaginalis*. The focus of these efforts, however, has largely been on women. More study is needed on the epidemiology of trichomoniasis in men as well as how to best diagnose and treat men who are infected, particularly given its high prevalence and communicability.
